# The Effect of Transverse Sinus Stenosis Caused by Arachnoid Granulation on Patients with Venous Pulsatile Tinnitus: A Multiphysics Interaction Simulation Investigation

**DOI:** 10.3390/bioengineering11060612

**Published:** 2024-06-15

**Authors:** Zhenxia Mu, Pengfei Zhao, Shifeng Yang, Lihui Zhuang, Heyu Ding, Xiaoyu Qiu, Bin Gao, Youjun Liu, Shusheng Gong, Guopeng Wang, Zhenchang Wang, Ximing Wang

**Affiliations:** 1 Department of Radiology, Shandong Provincial Hospital Affiliated to Shandong First Medical University, Jinan 250021, China; 2 Department of Radiology, Beijing Friendship Hospital, Capital Medical University, Beijing 100050, China; 3 Department of Biomedical Engineering, College of Chemistry and Life Science, Beijing University of Technology, Beijing 100124, China; 4 Department of Otolaryngology Head and Neck Surgery, Beijing Friendship Hospital, Capital Medical University, Beijing 100050, China

**Keywords:** pulsatile tinnitus, transverse sinus stenosis, arachnoid granulation, biomechanical, multiphysics interaction computation

## Abstract

This study aimed to investigate the effect of the transverse sinus (TS) stenosis (TSS) position caused by arachnoid granulation on patients with venous pulsatile tinnitus (VPT) and to further identify the types of TSS that are of therapeutic significance for patients. Multiphysics interaction models of six patients with moderate TSS caused by arachnoid granulation and virtual stent placement in TSS were reconstructed, including three patients with TSS located in the middle segment of the TS (group 1) and three patients with TTS in the middle and proximal involvement segment of the TS (group 2). The transient multiphysics interaction simulation method was applied to elucidate the differences in biomechanical and acoustic parameters between the two groups. The results revealed that the blood flow pattern at the TS and sigmoid sinus junction was significantly changed depending on the stenosis position. Preoperative patients had increased blood flow in the TSS region and TSS downstream where the blood flow impacted the vessel wall. In group 1, the postoperative blood flow pattern, average wall pressure, vessel wall vibration, and sound pressure level of the three patients were comparable to the preoperative state. However, the postoperative blood flow velocity decreased in group 2. The postoperative average wall pressure, vessel wall vibration, and sound pressure level of the three patients were significantly improved compared with the preoperative state. Intravascular intervention therapy should be considered for patients with moderate TSS caused by arachnoid granulations in the middle and proximal involvement segment of the TS. TSS might not be considered the cause of VPT symptoms in patients with moderate TSS caused by arachnoid granulation in the middle segment of the TS.

## 1. Introduction

Pulsatile tinnitus (PT) refers to a rhythmic noise that is felt in sync with the heartbeat in the absence of external stimuli, accounting for approximately 4%~10% of tinnitus [1,2]. Venous PT (VPT) is the most common type of PT, accounting for approximately 84% of PT, and is characterized by the reduction or elimination of noise when the ipsilateral internal jugular vein is compressed [3]. Persistent VPT has a negative impact on the quality of the patient’s life and may lead to depression or even suicide. The pathogenesis of VPT is multifactorial, and it is speculated that the noise’s sound source may be related to the venous blood flow caused by an abnormal venous vessel structure, such as transverse sinus (TS) stenosis (TSS), sigmoid sinus (SS) diverticulum (SSD), high jugular vein, and emissary vein [4]. The SS wall dehiscence (SSWD) around the venous vessel promotes the transmission of venous blood flow noise to the inner ear and auditory nerve, ultimately leading to VPT symptoms [5].

TSS mainly occurs at the middle segment of the TS and the entrance of the TS–SS junction, accompanied by SSD and SSWD [6]. The impact of high-velocity blood flow on the venous vessel at the TS–SS junction is considered to be a major pathogenic factor for SSD and SSWD formation [7]. Scholars have revealed that TSS is one of the most important pathological factors in the production of high-velocity blood flow [2]. Endovascular interventional therapy can effectively improve the abnormal hemodynamic patterns at the TS–SS junction by implanting stents at the TSS [8,9,10]. The diverticulum was minimized with new temporal bone remodeling after the operation [2]. These findings provide compelling evidence that TSS plays an important role in the occurrence and treatment of VPT. However, not all types of TSS can induce the production of VPT. The TSS characteristics may be benign or pathological. Patients with TSS accompanied by SSWD, with or without SSD, may directly induce the production of VPT [5]. TSS treatment is still contentious for patients with multiple vascular and temporal bone anomalies, and endovascular interventional therapy should be performed cautiously.

Previous research has demonstrated the significance of hemodynamics in the occurrence and treatment of VPT. Pereira found that TSS is the main causal factor behind the local hemodynamics variation in patients’ venous sinus [11]. Han discovered that the high-velocity jet blood flow formed in the distal segment of TSS enhances the impact on the venous vessels [2]. Tian found that the high-pressure load caused by blood flow increases the vibration of the blood vessel, which is transmitted to the inner ear and leads to VPT [5]. Our previous research discovered that stent implantation can effectively solve the VPT problem by blocking the impact of blood flow and reducing the pressure on the venous vessel [12]. Previous studies have provided new methods and ideas for the study of VPT. However, the present research has not elucidated the types of TSS that are therapeutic significance for VPT.

The purpose of this study was to investigate the effect of the TSS position caused by arachnoid granulation on patients with VPT and to identify the types of TSS that were meaningful for the occurrence and treatment of patients with VPT. Six patients with TSS at different positions caused by arachnoid granulation were retrospectively studied. Then, personalized multiphysics interaction models of the venous blood flow, venous vessel, temporal bone, and temporal bone air cell (TBAC) were reconstructed using computed tomography angiography (CTA) images. The biomechanical and acoustic characteristics of the different TSS positions and virtual stent implantation in TSS were compared using a multiphysics interaction simulation method. This study is of great significance in terms of elucidating the inducing mechanism of patients with VPT and selecting appropriate treatment strategies.

## 2. Materials and Methods

### 2.1. Image Data Acquisition

Six patients with VPT were retrospectively selected from a validated patient database. The CTA data collection was approved by the Institutional Review Board and conducted with each patient’s informed consent. All the patients were diagnosed with SSD, SSWD, TSS (the narrowest part of the TSS was caused by arachnoid particles pressing on the blood vessels), and/or high jugular bulb. The CTA data were obtained from a 256-slice spiral CT scanner (Brilliance, Philips Healthcare, GE Healthcare) using 512 × 512 image matrices, 0.625 mm slice thickness, 22 × 22 cm FOV, scanning the 6th cervical vertebra to the top of the skull, and including the bifurcation of bilateral common carotid arteries. Table 1 shows the patients’ information.

### 2.2. Model Reconstruction

**Figure 1:**
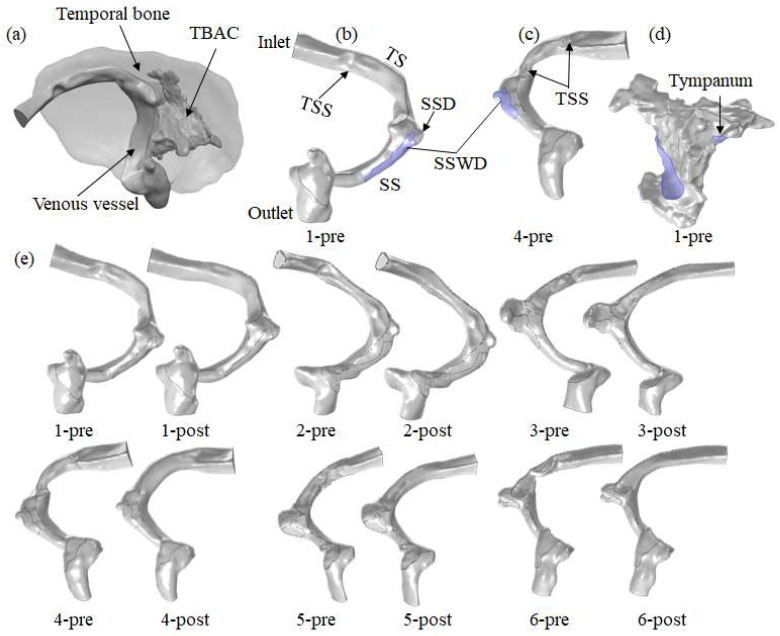
Geometry models. (**a**) Position of the temporal bone, venous vessel and TBAC. Taking patient 1–pre and patient 4–pre as examples: (**b**,**c**) position of the inlet, TS, TSS, SSD, SSWD, SS and outlet. (**d**) Position of the tympanum in TBAC. (**e**) Preoperative and postoperative venous vessel geometry models of each patient.

The three-dimensional geometries were reconstructed from the CTA data using Mimics 20.0 (Materialise, Leuven, Belgium) and smoothed with Geomagic 2024 (Geomagic, Houston, TX, USA). Each patient’s geometric models included the venous fluid model, vessel model, temporal bone model, and TBAC model (Figure 1a–d). The venous fluid model was reconstructed from the starting segment of the TS to the distal of the SS (Figure 1b,c). The vessel wall model, with a thickness of 0.5 mm, was extracted from the venous fluid model [5]. The SSD domain was characterized as the projecting domain at the TS–SS junction. The SSWD domain was defined as the region where the vessel wall intersected with the TBAC domain as a result of temporal bone dehiscence.

Patients 1 to 3 were classified as having intrinsic stenosis in the middle segment of the TS caused by large arachnoid granulation, and they were designated as group 1. The stenosis length ranged from 5 to 7 mm. Patients 4 to 6 were classified as being in the middle segment of the TS with proximal involvement caused by multiple continuous arachnoid granulations, and they were categorized as group 2. The stenosis length was larger than 10 mm. Additionally, patients 4 and 6 had extrinsic stenosis. The cross-sectional area of the TS at the most stenosed region was assessed, as well as the normal cross-sectional area of the TS at the stenosed distal segment. The stenosis rate of each patient was calculated by dividing the cross-sectional area of the TS at the most stenosed region by the normal cross-sectional area of the TS. The most severe TSS location among all the patients was identified as intrinsic stenosis caused by arachnoid granulation compression. The degree of TSS in both groups of patients was within the range of moderate stenosis (Table 2).

Freeform Plus 17.0 (3D Systems, Freeform, Cary, NC, USA) software was used to reconstruct the model after stent placement for TSS treatment. Six patients with TSS were classified as patients 1 to 6–pre, respectively (Figure 1e). After virtual surgical treatment, six patients without TSS were classified as patients 1 to 6–post, respectively (Figure 1e). Table 2 displays the geometrical features and information.

### 2.3. Governing Equations

The governing equation for the venous fluid domain was found by substituting the convection velocity with the relative velocity of the moving mesh in the Navier–Stokes equation. In the ALE formulation of the Navier–Stokes equation, the continuity and momentum governing equations of the venous fluid domain are presented as Equations (1) and (2) [13]:

(1) $$\rho_{f}\frac{\partial v_{f}}{\partial t}+\rho_{f}\left(v_{f}\cdot \nabla \right)v_{f}=\nabla \cdot \left[-p_{f}I+\mu \left(\nabla v_{f}+{\left(\nabla v_{f}\right)}^{T}\right)\right]+F_{f}$$

(2) $$\nabla \cdot v_{f}=0$$

where $\rho_{f}$ is the fluid density, $p_{f}$ is the fluid pressure, μ is the fluid viscosity, $v_{f}$ is the fluid velocity vector, $F_{f}$ is the body force (per unit volume) acting on the fluid domain, and I is the fluid unit tensor.

The solid domains of the vessel and temporal bone are governed by Newton’s second law, as depicted in Equation (3) [14]:

(3) $$\rho_{s}\frac{\partial^{2}x_{s}}{\partial t^{2}}=\nabla \cdot \sigma_{s}+F_{s}$$

where $\rho_{s}$ is the solid density, $x_{s}$ is the solid displacement, $F_{s}$ is the force vector (per unit volume) acting on the solid domain, and $\sigma_{s}$ is the solid stress tensor. The $\sigma_{s}$ is given as Equation (4) [14]:

(4) $$\sigma_{s}=2\mu_{S}\epsilon +\lambda_{s}tr(\epsilon )I$$

where $\lambda_{s}$ and $\mu_{S}$ are the first- and second-order Lamé parameters, respectively. ϵ is the strain tensor, I is the characteristics matrix, and the word ‘tr’ denotes the trace function. $\lambda_{s}$ and $\mu_{S}$ are given as Equations (5) and (6) [14]:

(5) $$\lambda_{s}=\frac{\upsilon E}{\left(1+\upsilon \right)\left(1-2\upsilon \right)}$$

(6) $$\mu_{S}=\frac{E}{2\left(1+\upsilon \right)}$$

where E is the solid elastic module, and υ is the Poisson’s ratio.

The fluid–structure interaction interface in this study is the interface between the venous blood fluid domain and the solid domains of the venous blood vessel, which needs to follow the basic conservation principle. The dynamic and kinematic condition equations are shown in Equations (7) and (8) [15]:

(7) $$\tau_{f}\cdot n_{f}=\tau_{s}\cdot n_{s}$$

(8) $$d_{f}=d_{s}$$

where $\tau_{f}$ and $\tau_{s}$ are the stress tensor of fluid and solid at the interface, respectively. $d_{f}$ is the fluid displacement at the interface, $d_{s}$ is the solid displacement at the interface, and $n_{f}$ and $n_{s}$ are the fluid and solid normal vector at the interface, respectively.

The acoustic simulation is governed by the following wave equation, ignoring the air viscosity and heat exchange, as depicted in Equation (9) [16]:

(9) $$\frac{1}{c_{0}^{2}}\frac{\partial^{2}p}{\partial t^{2}}-\nabla^{2}p=0$$

where $c_{0}$ is the acoustic velocity of adiabatic air, and p is the sound pressure.

The fluid–structure and acoustic–structure interaction studies were solved using COMSOL Multiphysics software 5.6 (COMSOL AB, Stockholm, Sweden). The fully coupled method using the MUMPS direct solver was applied to solve the fluid–structure interaction problem. The maximum number of iterations was set as 50, and the automatic time step was chosen to meet the CFL criterion <1. The iterative convergence criteria were based on a threshold of residuals less than 10^−3^. The one-way coupled technique with the suggested direct solver from MUMPS was selected to solve the acoustic–structure interaction problem. The maximum iteration number was set as 25, and the convergence precision was set as less than 10^−3^.

### 2.4. Boundary Condition and Calculation Setting

**Figure 2:**
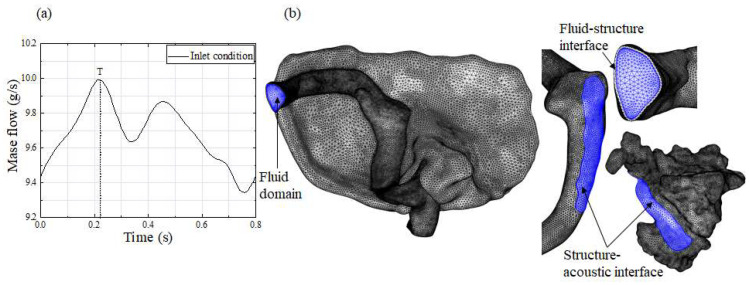
(**a**) Tetrahedral mesh of each domain model. (**b**) Boundary condition of the inlet section.

In this study, the transient pulsating mass flow waveform was applied as the boundary condition of the inlet section (Figure 2a) [5]. A pressure of 0 Pa was applied to the outlet section.

The venous blood fluid was assumed to be homogenous, incompressible, and Newtonian ($\rho_{f}\;$ = 1050 kg/m^3^, μ  = 0.0035 Pa∙s) [17]. The maximum Reynolds number at the maximum velocity moment was calculated below 2300 (Figure 2a, T) [18]. The venous blood fluid was considered to be laminar. The venous vessel and temporal bone solid structures were set as an isotropic linear elastic material. The venous vessel density was taken as 1.05 g/cm^3^, the elastic module was 1.26 MP, and the Poisson’s ratio was 0.3 [19,20]. The temporal bone density was taken as 2.00 g/cm^3^, the elastic module was 12,000 MP, and the Poisson’s ratio was 0.3 [21]. The lateral surface of the vessel wall was in contact with rigid osseous tissue (such as endocranium), while the medial surface was attached to the temporal bone. Thus, the inlet section, outlet section, and lateral surface of the vessel wall were all fixed [5]. The penalty function was utilized to define the contact between the medial surface of the vessel wall and the temporal bone. The cardiac cycle time was fixed to 0.8 s. To eliminate the effect of the calculation initialization on the results and improve the convergence of the calculation, four cardiac cycles were computed for each patient, and the fluid–structure interaction results from the last cardiac cycle were extracted for analysis.

The displacement of the vessel wall in the SSWD domain extracted from the fluid–structure interaction research was applied as the input condition for the acoustic–structure interaction. The venous blood noise was supposed to be only transmitted through the SSWD domain. The air density of the TBAC component was taken as 1.139 kg/m^3^, with a sound speed of 340 m/s [16]. The temporal bone edge in contact with the TBAC was set as the acoustic impedance surface with an impedance of 5.57 MP Pa∙s/m [22]. The physiological tympanum of a human was set as the receiving boundary of venous blood sound with an impedance model, as described in Equation (10) [22,23]:

(10) $$-n\left[-\frac{1}{\rho }\left(\nabla p_{t}-q_{d}\right)\right]=\frac{1}{Z_{i}}\frac{\partial p_{t}}{\partial t}$$

where ρ denotes the air density, $p_{t}$ is the total pressure, $q_{d}$ is the domain volumetric, and $Z_{i}$ represents the specific acoustic input impedance of the external domain.

### 2.5. Mesh Generation

The computational tetrahedral grid was built using COMSOL 5.6 (COMSOL AB, Stockholm, Sweden). Mesh refinement experiments were carried out during the transient simulation with the multiphysics interaction model to establish the mesh-independent simulation (Figure 2b). The average displacement of the vessel wall in the SSWD domain was utilized to estimate the optimum grid number for the fluid–structure interaction simulation. Similarly, the average sound pressure level at the tympanum was utilized to establish the appropriate grid number for the acoustic–structure interaction simulation. When the highest relative inaccuracy of the average displacement and sound pressure level between the fine and coarse meshes was less than 5%, the meshes were deemed satisfactory in this study. The fine mesh number of the venous blood fluid, vessel wall, and TBAC was around 0.99, 0.19, and 0.26 million. The details of the grid were consistent with previous research [18]. The maximum element size used in the fluid–structure and acoustic–structure interaction simulation was 0.3 mm. The wedge-shaped two-boundary-layer mesh was created in the venous blood fluid domain.

### 2.6. Hemodynamic Analysis

The blood flow velocity vector, wall pressure distribution, average wall pressure ($P_{\mathrm{avg}}$) in the SSWD domain, average displacement ($D_{\mathrm{avg}}$) of the vessel wall in the SSWD domain, and average sound pressure level (${SPL}_{\mathrm{avg}}$) at the tympanum were calculated to evaluate the differences in the biomechanical and acoustic characteristics of different TSS positions and virtual stent implantation in TSS. The expressions of $P_{\mathrm{avg}}$, $D_{\mathrm{avg}}$, and ${SPL}_{\mathrm{avg}}$ are shown as follows.

$P_{\mathrm{avg}}$ represents the average value of the wall pressure in the SSWD domain, which is defined as Equation (11):

(11) $$P_{\mathrm{avg}}=\frac{\sum P_{i}\cdot area_{i}}{\sum area_{i}}$$

where $P_{\mathrm{avg}}$ represents the average wall pressure. $P_{i}$ is the pressure value of element i, $area_{i}$ is the area of element i.

$D_{\mathrm{avg}}$ represents the average displacement value of the vessel wall in the SSWD domain, indicating the overall displacement in the area. The higher the value, the stronger the vibration of the blood vessel wall. $D_{\mathrm{avg}}$ is defined as Equation (12):

(12) $$D_{\mathrm{avg}}=\frac{\sum D_{i}\cdot area_{i}}{\sum area_{i}}$$

where $D_{\mathrm{avg}}$ represents the average displacement. $D_{i}$ is the displacement value of element i.

${SPL}_{\mathrm{avg}}$ represents the average value of the sound pressure level at the tympanum. The value of ${SPL}_{\mathrm{avg}}$ reflects the intensity of the perceived noise. ${SPL}_{\mathrm{avg}}$ is defined as Equation (13):

(13) $${SPL}_{\mathrm{avg}}=\frac{\sum {SPL}_{i}\cdot area_{i}}{\sum area_{i}}$$

where ${SPL}_{\mathrm{avg}}$ represents the average sound pressure level. ${SPL}_{i}$ is the displacement value of element i.

## 3. Results

### 3.1. Velocity Field

**Figure 3:**
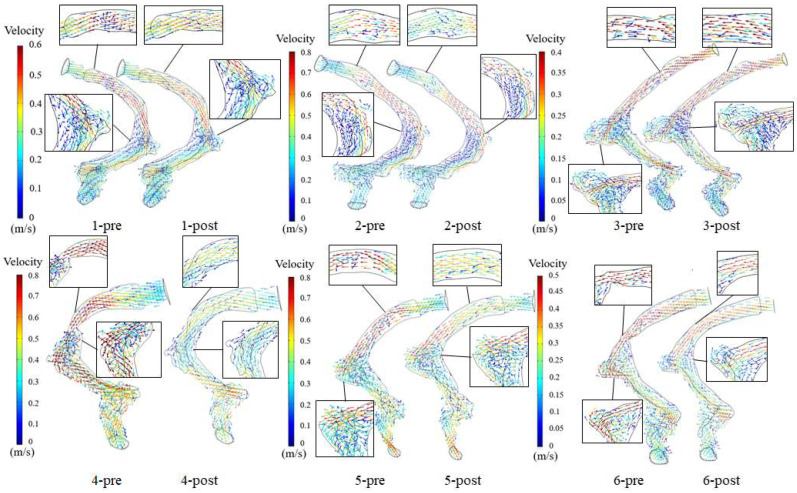
Preoperative and postoperative velocity vector at the maximum velocity moment of each patient.

Figure 3 depicts the preoperative and postoperative velocity vector at the maximum velocity moment for each patient. The vortex formed at the TS–SS junction and SSD region. The blood flow velocity of the postoperative patients decreased at the TSS region compared with the preoperative patients. At the TS–SS junction region, the blood flow velocity of group 1 (patients 1 to 3) was similar to the preoperative state. Nevertheless, the blood flow velocity of group 2 (patients 4 to 6) was lower than the preoperative state.

### 3.2. Wall Pressure

**Figure 4:**
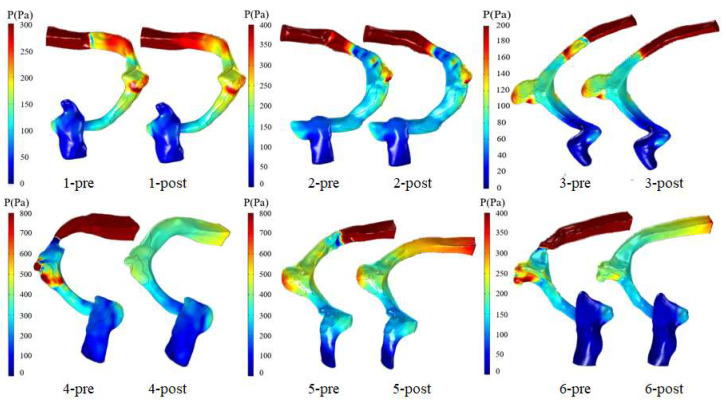
Preoperative and postoperative wall pressure at the maximum velocity moment of each patient.

Figure 4 describes the preoperative and postoperative wall pressure distribution at the maximum velocity moment for each patient. In the preoperative patients, the high wall pressure was distributed in the region where the blood flow impinged on the vessel wall and SSD region. In the postoperative patients, the high wall pressure distribution of group 1 (patients 1 to 3) was consistent with the preoperative distribution characteristics. The high wall pressure distribution in the region where the blood flow impinged on the vessel wall and SSD region of group 2 (patients 4 to 6) was decreased.

**Figure 5:**
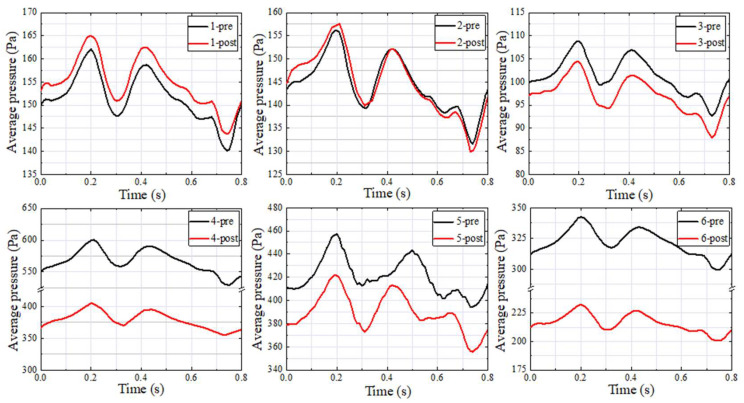
Preoperative and postoperative average wall pressure in the SSWD domain in the whole cardiac cycle of each patient.

Figure 5 illustrates the preoperative and postoperative $P_{\mathrm{avg}}$ in the SSWD domain in the whole cardiac cycle for each patient. In group 1 (patients 1 to 3), there was little difference between the postoperative $P_{\mathrm{avg}}$ in the whole cardiac cycle and preoperative state. In contrast, the postoperative $P_{\mathrm{avg}}$ in the whole cardiac cycle of group 2 (patients 4 to 6) was decreased. Table 3 shows the preoperative and postoperative $P_{\mathrm{avg}}$ at the maximum velocity moment in the SSWD domain for each patient. The $P_{\mathrm{avg}}$ difference was estimated by subtracting the postoperative $P_{\mathrm{avg}}$ from the preoperative $P_{\mathrm{avg}}$. The $P_{\mathrm{avg}}$ of group 1 (patients 1 to 3) was decreased by −3.33, −1.90, and 4.68 Pa, respectively. The $P_{\mathrm{avg}}$ of group 2 (patients 4 to 6) was decreased by 196.69, 50.39, and 110.44 Pa, respectively.

### 3.3. Vessel Wall Displacement

**Figure 6:**
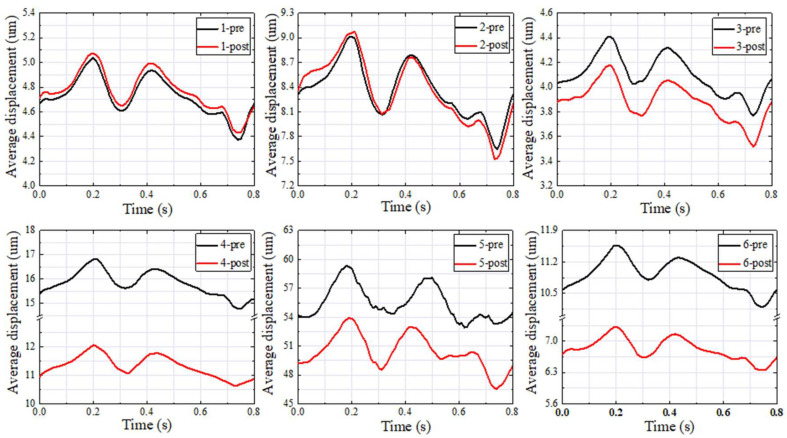
Preoperative and postoperative average displacement of the venous vessel in the SSWD domain in the whole cardiac cycle of each patient.

Figure 6 describes the preoperative and postoperative $D_{\mathrm{avg}}$ of the vessel wall in the SSWD domain in the whole cardiac cycle for each patient. In group 1 (patients 1 to 3), the postoperative $D_{\mathrm{avg}}$ of the vessel wall in the SSWD domain during the cardiac cycle was basically the same as that of the preoperative state. In contrast, in group 2 (patients 4 to 6), the postoperative $D_{\mathrm{avg}}$ of the vessel wall in the SSWD domain during the cardiac cycle was decreased significantly.

Table 3 depicts the preoperative and postoperative $D_{\mathrm{avg}}$ of the vessel wall in the SSWD domain at the maximum velocity moment for each patient. The $D_{\mathrm{avg}}$ difference was estimated by subtracting the postoperative $D_{\mathrm{avg}}$ from the preoperative $D_{\mathrm{avg}}$. The $D_{\mathrm{avg}}$ of group 1 (patients 1 to 3) was decreased by −0.04, −0.09, and 0.24 um, respectively. The $D_{\mathrm{avg}}$ of group 2 (patients 4 to 6) was decreased by 4.80, 7.17, and 4.27 um, respectively.

### 3.4. Sound Pressure Level

Table 3 depicts the preoperative and postoperative ${SPL}_{\mathrm{avg}}$ at the tympanum with the first mode frequency for each patient. The ${SPL}_{\mathrm{avg}}$ difference was calculated by subtracting the preoperative ${SPL}_{\mathrm{avg}}$ from the postoperative ${SPL}_{\mathrm{avg}}$. In group 1 (patients 1 to 3), the ${SPL}_{\mathrm{avg}}$ of the postoperative patients was decreased by −0.30, −1.02, and −0.32 dB, respectively. In group 2 (patients 4 to 6), the ${SPL}_{\mathrm{avg}}$ of the postoperative patients was decreased by 9.27, 11.25, and 18.40 dB, respectively.

## 4. Discussion

Previous researchers focused on the association between the TSS features and VPT events, as well as the effect of the blood flow on the SS region in patients. In this study, a multiphysics interaction method combining fluid–structure–acoustics was applied to elucidate the effect of the TSS position caused by arachnoid granulation on patients with VPT, as well as the types of TSS that have therapeutic significance for VPT. By comparing numerical simulations of the TSS positions and virtual stent treatments, it was revealed that the blood flow pattern at the TS–SS junction with different stenosis positions changed significantly. The middle segment and involvement of the proximal TSS caused by multiple continuous arachnoid granulations resulted in a jet blood flow impacting the TS–SS junction vessel wall, which enhanced the vibration of the vessel wall. The vibration of the vessel wall was transmitted through the TBAC, resulting in an increase in the sound pressure level at the tympanic. Patients with moderate TSS whose stenosis was located in the middle segment of the TS with proximal involvement caused by multiple continuous arachnoid granulation might be considered for TSS treatment. Patients with moderate TSS whose stenosis was located in the middle segment of the TS caused by arachnoid granulation indicated that TSS might not be the cause of VPT.

Han found that the blood velocity was higher at the TSS, and the downstream region of the TSS showed increased twisting and curling [8]. The blood flow pattern at the TSS in this study was consistent with Han’s work. Our previous study using hemodynamic and 4D flow MRI methods found that high wall pressure in the SSWD domain was one of the causes of VPT. Due to individual differences among patients, the $P_{\mathrm{avg}}$ in the SSWD domain was distributed in the range of 150~600 Pa [7]. In this study, the position and range of the high $P_{\mathrm{avg}}$ in the SSWD domain were consistent with previous studies (Table 3). By comparing the geometric model features, the ${SPL}_{\mathrm{avg}}$ at the tympanum with the first mode frequency (patient 4-post, 58.08 dB) was similar to that in Tian’s work (56.9 dB) [5,16].

Researchers generally believe that the changes in the blood flow patterns at the TS–SS junction caused by TSS are among the causes in patients with VPT [11,17,24]. By comparing the simulation results of patients with TSS before and after stent treatment, this study discovered that the compression of multiple continuous arachnoid granulations on the TS vessel disrupted the laminar flow state in the TS, resulting in the high-velocity jet entering the TS–SS junction and impacting the vessel wall, forming a vortex at the TS–SS junction (Figure 3). The impact of the high-velocity jet on the vessel wall contributed to the blood vessel wall pressure increasing, and the vibration of the vessel wall was amplified under the high wall pressure (Figure 1, Figure 2 and Figure 3). Subsequently, the vibration of the vessel wall was transmitted to the tympanic through the TBAC, causing an increase in the sound pressure level of the tympanic (Table 3). The stent placement at the TSS could effectively weaken the impact of the high-velocity jet on the vessel wall at the TS–SS junction [8]. Furthermore, the wall pressure and vessel wall vibration could be lowered to alleviate VPT symptoms.

Huang’s research showed that a significant VPT treatment effect could be achieved by the $P_{\mathrm{avg}}$ decreasing by 20.07 Pa under the condition that the diverticulum was returned to 60% [25]. Through numerical simulation of personalized models with different positions of the TSS, this study found that the maximum $P_{\mathrm{avg}}$ reduction in group 1 was 4.68 Pa (0.04%), the minimum $P_{\mathrm{avg}}$ reduction in group 2 was 50.39 Pa (10.73%), and the maximum $P_{\mathrm{avg}}$ reduction was 196.69 Pa (32.74%). Thus, the jet blood flow from the middle segment of the TSS caused by arachnoid granulation had a very weak impact on the vessel wall at the TS–SS junction. In contrast, the jet blood flow from the middle segment involving the proximal segment of the TSS caused by arachnoid granulation had a severe impact on the vessel wall.

SSWD reconstruction surgery is considered to be the most important, safe, and effective treatment method for patients with VPT. However, this approach does not provide a desirable therapeutic result for all patients [26,27]. For patients with TSS and SSWD, traditional SSWD reconstruction only solves the problems of SSWD [28]. The high-velocity jet blood flow caused by residual TSS continuously impacts the reconstructed bone wall, resulting in the recurrence of VPT [2,26]. The results of the $P_{\mathrm{avg}}$ difference revealed that endovascular interventional therapy for patients with the middle segment involving the proximal segment of the TSS caused by arachnoid granulation could improve the hemodynamics of the TS–SS junction. The degree of $P_{\mathrm{avg}}$ reduction was within the effective treatment range of SSWD reconstruction surgery [29]. The results of the ${SPL}_{\mathrm{avg}}$ reduction (maximum ${SPL}_{\mathrm{avg}}$ reduction of 18.40 dB and minimum ${SPL}_{\mathrm{avg}}$ reduction of 9.27dB) at the tympanic demonstrated that this method might effectively alleviate VPT symptoms [30]. Therefore, endovascular interventional therapy should be considered for patients with the middle segment involving the proximal segment of the TSS to reduce the possibility of recurrence of VPT symptoms.

There are some limitations to this study. Firstly, the number of samples was small and only VPT patients with moderate TSS were evaluated. It is necessary to expand the sample and type of patients. To cover all types of patients with TSS, future studies will include patients with mild and severe TSS to further enhance the evidence of the analysis. Secondly, endovascular interventional therapy was a virtual simulation study without considering the influence of stent construction on the vessel wall. Thirdly, since the specific blood flow velocity of the patients was unavailable, the velocity data in the literature were used for the numerical simulation [5]. Finally, the venous vessels were surrounded by hard tissues such as the temporal bone and endocranium. The venous vessel wall was assumed to be a linear elastic material. Future research will focus on patient cohort studies and obtain personalized blood flow velocity data for patients with VPT through 4D flow magnetic resonance imaging technology. Moreover, a personalized stent model suitable for venous vessels will be designed to explore the interaction between blood vessels and stents. The material properties of the repair materials will be obtained through in vitro experiments to further improve the accuracy of the study.

## 5. Conclusions

The jet blood flow caused by the arachnoid granulation in the middle segment involving the proximal segment of the TS enhanced the impact on the vessel wall at the TS–SS junction, leading to an increase in the vessel wall pressure. The periodic pulsation of the high wall pressure promoted an increase in the venous vessel wall vibration, which was transmitted to the tympanic through the TBAC, causing the sound pressure level to increase. Moderate TSS in the middle segment of the TS caused by arachnoid granulation might not be considered as the cause of VPT symptoms. Moderate TSS in the middle segment involving the proximal segment of the TS caused by multiple continuous arachnoid granulations should be considered as the cause of VPT symptoms and intravascular interventional therapy should be considered.

## Figures and Tables

**Table 1:** Patients’ information.

Patients	Age	Gender	Side	Diagnosis
1	41	Female	Left	SSWD+SSD+TSS+HJB
2	26	Female	Left	SSWD+SSD+TSS
3	55	Female	Right	SSWD+SSD+TSS
4	61	Female	Right	SSWD+SSD+TSS+HJB
5	53	Male	Right	SSWD+SSD+TSS
6	58	Female	Right	SSWD+SSD+TSS+HJB

Sigmoid sinus wall dehiscence (SSWD); sigmoid sinus diverticulum (SSD); transverse sinus stenosis (TSS); high jugular bulb (HJB).

**Table 2:** Geometrical features and details.

Patients	Inlet Area (mm^2^)	Outlet Area (mm^2^)	TSS Rate (%)	TSS Position	SSWD Area (mm^2^)	TBAC Volume (mm^3^)	Tympanum Area (mm^2^)
1	40.73	60.74	65.11	Middle	129.02	5778.50	16.74
2	51.31	42.31	57.93	Middle	221.97	6517.60	63.66
3	22.80	58.50	60.94	Middle	140.74	8707.00	16.14
4	73.77	52.45	67.62	Middle and proximal	90.25	7638.00	29.73
5	64.00	36.64	62.43	Middle and proximal	98.15	4393.60	13.01
6	34.34	55.75	62.34	Middle and proximal	30.75	6855.20	15.49

**Table 3:** Preoperative and postoperative biomechanical and acoustic characteristics of each patient.

Patients	P_avg_ (Pa)	D_avg_ (um)	SPL_avg_ (dB)	P_avg_ Difference (Pa)	D_avg_ Difference (um)	SPL_avg_ Difference (dB)
1–pre	161.34	5.02	69.63	−3.33	−0.04	−0.30
1–post	164.67	5.06	69.93			
2–pre	155.72	8.99	78.89	−1.90	−0.09	−1.02
2–post	157.62	9.08	79.90			
3–pre	108.32	4.39	62.42	4.68	0.24	−0.32
3–post	103.64	4.15	62.74			
4–pre	600.71	16.83	67.35	196.69	4.80	9.27
4–post	404.02	12.03	58.08			
5–pre	469.67	60.78	96.05	50.39	7.17	11.25
5–post	419.27	53.61	84.80			
6–pre	341.92	11.56	59.72	110.44	4.27	18.40
6–post	231.48	7.29	41.32			

## Data Availability

The original data are available from the corresponding author upon appropriate request.

## Nomenclature

CTA	computed tomography angiography
VPT	venous pulsatile tinnitus
SS	sigmoid sinus
TSS	transverse sinus stenosis
SSD	sigmoid sinus diverticulum
SSWD	sigmoid sinus wall dehiscence
TBAC	temporal bone air cell
